# Liquid-Liquid extraction of phenolic compounds in systems based on acetonitrile + water + polyvinylpyrrolidone at 298.15 K

**DOI:** 10.1016/j.dib.2018.09.067

**Published:** 2018-09-27

**Authors:** Bruno Sales Oliveira, Camila Maria de Souza D’Anzicourt, Cleide Mara Faria Soares, Ranyere Lucena de Souza, Álvaro Silva Lima

**Affiliations:** a Tiradentes University, Av. Murilo Dantas 300, Farolândia, 49032-490 Aracaju, SE, Brazil; b Institute of Technology and Research, Av. Murilo Dantas, 300-Prédio do ITP, Farolândia, 49032-490 Aracaju, SE, Brazil

## Abstract

This paper contains data related to the research paper entitled “Organic two-phase system based on acetonitrile + water + polyvinylpyrrolidone, a novel concept of liquid–liquid equilibrium: phase diagrams and phenolic compounds partitioning”. Data of phase equilibrium were obtained using the cloud point method. After this step, some blending points were chosen to perform the phenolic compounds partitioning (gallic acid, quercetin dihydrate and cyanidin 3-O-glucoside chloride)

## Specifications table

**Table:** 

Subject area	*Chemical Engineering*
More specific subject area	*Separation Science.*
Type of data	*Table and figure*
How data was acquired	*Liquid–liquid equilibrium data were determined gravimetrically at 298.15 *±* 1.00* *K and 0.10* ± *0.01* *MPa using the cloud point method*
Data format	*Raw* (Table DB1, Table DB2, Table DB3, Table DB4, Table DB5, Table DB6) *and analyzed (*Table DB7*and*Fig. DB1, Fig. DB2, Fig. DB3, Fig. DB4)
Experimental factors	*Calculations performed by mass balance*
Experimental features	*Mass balance*
Data source location	*Aracaju-Sergipe, Brail*
Data accessibility	*With this article.*
Related research article	*Organic two-phase system based on acetonitrile + water + polyvinylpyrrolidone, a novel concept of liquid–liquid equilibrium: phase diagrams and phenolic compounds partitioning*

## Value of the data

• The liquid–liquid equilibrium data for the proposed systems are not yet presented in the literature.
• The phase equilibrium data shows that the phases are rich in acetonitrile, so the systems are called biphasic organic
• The data can help in the choice of mixing points for the partitioning of phenolic compounds.
• The data of partition coefficients allow the application of the proposed systems in the purification and/or the concentration of phenolic compounds.

## 1 Data

**Fig. DB1:**
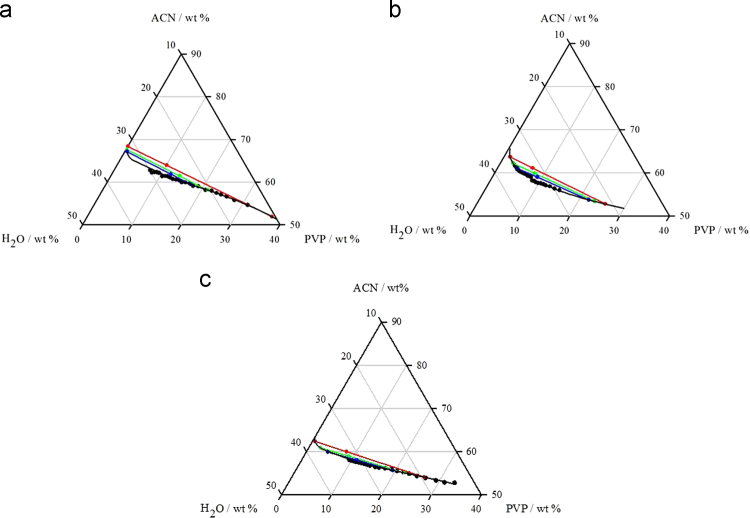
Phase diagrams for ternary systems composed of acetonitrile + PVP + water at 298.15 K and 0.1 MPa. (a) PVP 10,000 g mol^−1^; (b) PVP 29,000 g mol^−1^; (c) PVP 40,000 g mol^−1^.

Table DB1, Table DB2, Table DB3 depicted the equilibrium data for systems formed by polyvinylpyrrolidone – PVP (1), acetonitrile – ACN (2) and water – H_2_O (3), obtained at 298.15 K and 0.1 MPa, while the Fig. DB1 presents the triangular diagram for liquid–liquid equilibrium of the systems.

**Table DB1:** Experimental binodal mass fraction data for the system composed of PVP 10,000 g mol^−1^ (1) + ACN (2) + water (3) at 298.15 K and 0.1 MPa.^a^

**PVP**	**ACN**	**H**_**2**_**O**
**100*w***_**1**_	**100*w***_**2**_	**100*w***_**3**_
31.18	54.64	14.18
27.91	55.77	16.32
25.90	56.65	17.45
24.56	57.09	18.35
23.49	57.43	19.08
22.24	57.91	19.84
20.87	58.13	21.00
19.01	59.00	21.99
17.90	59.09	23.00
16.97	59.68	23.36
15.89	59.90	24.20
15.17	59.92	24.91
14.44	60.45	25.12
13.69	60.56	25.75
12.97	60.71	26.33
12.20	61.05	26.75
11.52	61.39	27.08
11.07	61.40	27.53
10.48	61.35	28.16
9.51	62.10	28.38
8.90	62.33	28.76
8.49	62.32	29.18
7.94	62.23	29.82

a Standard uncertainties *u* are *u*([PVP], [ACN] or [H_2_O]) = 0.01, *u*(*T*) = 1 K, and *u*(*p*) = 10 kPa.

**Table DB2:** Experimental binodal mass fraction data for the system composed of PVP 29,000 g mol^−1^ (1) + ACN (2) + water (3) at 298.15 K and 0.1 MPa.^a^

**PVP**	**ACN**	**H**_**2**_**O**
**100*w***_**1**_	**100*w***_**2**_	**100*w***_**3**_
14.98	55.97	29,05
13.78	56.37	29,85
12.61	56.86	30,54
11.66	57.12	31,22
11.01	57.40	31,59
10.43	57.56	32,01
9.85	57.62	32,53
9.31	57.85	32,84
8.87	58.08	33,05
8.39	58.24	33,37
7.74	59.01	33,25
7.31	59.24	33,44
6.95	59.34	33,71
6.54	59.60	33,86
6.08	59.81	34,11
5.68	59.98	34,34
5.29	60.16	34,54
5.02	60.24	34,74
4.72	60.45	34,83
4.42	60.65	34,93
4.17	60.76	35,07
3.98	60.96	35,06
3.74	61.33	34,94
3.56	61.38	35,06
3.40	61.55	35,06
3.26	61.64	35,10
3.13	61.75	35,12
2.97	61.98	35,04

a Standard uncertainties *u* are *u*([PVP], [ACN] or [H_2_O]) = 0.01, *u*(*T*) = 1 K, and *u*(*p*) = 10 kPa.

**Table DB3:** Experimental binodal mass fraction data for the system composed of PVP 40,000 g mol^−1^ (1) + ACN (2) + water (3) at 298.15 K and 0.1 MPa.^a^

**PVP**	**ACN**	**H**_**2**_**O**
**100*w***_**1**_	**100*w***_**2**_	**100*w***_**3**_
33.25	52.77	13.98
31.16	5287	15.97
29.25	53.33	17.42
26.88	53.88	19.24
24.90	54.36	20.74
23.06	54.92	22.01
21.76	55.23	23.01
20.36	55.48	24.15
19.04	55.98	24.99
17.59	56.16	26.24
16.31	56.33	27.36
15.46	56.48	28.06
14.27	56.79	28.94
13.65	56.98	29.37
13.09	57.16	29.75
12.57	57.28	30.14
12.29	57.40	30.31
11.82	57.51	30.67
11.34	57.64	31.02
10.97	57.68	31.35
10.59	57.79	31.62
10.19	57.90	31.91
9.3	58.00	32.17
9.52	58.11	32.37

a Standard uncertainties *u* are *u*([PVP], [ACN] or [H_2_O]) = 0.01, *u*(*T*) = 1 K, and *u*(*p*) = 10 kPa.

In order to characterize the phases, The Table DB4, Table DB5DB6 show the density and viscosity of both phases for systems based on PVP + ACN + H_2_O at 298.15 K and 0.1 MPa.

**Table DB4:** Experimental density (g cm^-3^) and viscosity (mPa s) of organic two-phase system used in the partition of biomolecules. System A consists of 12.05 wt% PVP (10,000 g mol^−1^) + 62.05 wt% ACN + 25.9 wt% H_2_O at 298.15 K and 0.1 MPa.^a^

Temperature K	Density/g cm^3^		Viscosity/mPa s	
	Top phase	Bottom phase	Top phase	Bottom phase
278	0.8512	1.0243	0.65450	82.1320
283	0.8465	1.0240	0.60373	54.2160
288	0.8416	1.0250	0.55202	39.1010
293	0.8367	1.0228	0.50468	30.0560
298	0.8318	1.0197	0.46222	24.6370
303	0.8268	1.0157	0.42625	20.4290
308	0.8217	1.0121	0.39301	17.1210
313	0.8166	1.0081	0.36319	14.4770
318	0.8114	1.0039	0.33684	12.3300
323	0.8062	0.9997	0.31249	10.5560
328	0.8010	0.9958	0.29140	9.1172
333	0.7956	0.9914	0.27046	7.9359
338	0.7902	0.9864	0.24036	6.9630
343	0.7847	0.9819	0.19725	6.1695

a Standard uncertainties *u* are *u*(*ρ*)= 5× 10^-4^ g cm^-3^, *u*(*η*) = 0.35%, *u*(*T*) = 0.02 K, and *u*(*p*) = 10 kPa.

**Table DB5:** Experimental density (g cm^-3^) and viscosity (mPa s) of organic two-phase system used in the partition of biomolecules. System B consists of 9.02 wt% PVP (29,000 g mol^−1^) + 59.02 wt% ACN + 31.96 wt% H_2_O at 298.15 K and 0.1 MPa.^a^

Temperature K	Density/g cm^3^		Viscosity/mPa s	
	Top phase	Bottom phase	Top phase	Bottom phase
278	0.8620	1.0125	0.74113	103.490
283	0.8574	1.0101	0.67609	79.979
288	0.8528	1.0097	0.61341	58.963
293	0.8481	1.0071	0.55958	46.305
298	0.8434	1.0037	0.50991	38.022
303	0.8385	1.0002	0.46653	31.952
308	0.8336	0.9965	0.42614	27.037
313	0.8287	0.9924	0.38824	23.060
318	0.8237	0.9879	0.35063	19.826
323	0.8186	0.9834	0.31284	17.142
328	0.8138	0.9784	0.27960	14.879
333	0.8086	0.9727	0.26509	12.979
338	0.8034	0.9658	0.23186	11.358
343	0.7980	0.9563	0.21122	9.880

a Standard uncertainties *u* are *u*(*ρ*) = 5 × 10^-4^ g cm^−3^, *u*(*η*) = 0.35%, *u*(*T*) = 0.02 K, and *u*(*p*) = 10 kPa.

**Table DB6:** Experimental density (g cm^−3^) and viscosity (mPa s) of organic two-phase system used in the partition of biomolecules. In system C we used PVP 40,00 g mol^-1^ + 58.89% ACN + 32.22% H_2_O at 298.15 K and 0.1 MPa.^a^

Temperature K	Density/g cm^−3^		Viscosity/mPa s	
	Top phase	Bottom phase	Top phase	Bottom phase
278	0.8628	1.0004	0.76526	156.8400
283	0.8582	0.9984	0.69710	113.1300
288	0.8536	0.9966	0.63041	84.6880
293	0.8489	0.9942	0.57318	68.5900
298	0.8441	0.9914	0.52248	56.2750
303	0.8392	0.9884	0.47817	47.0050
308	0.8343	0.9851	0.43868	40.0810
313	0.8294	0.9808	0.40337	34.4360
318	0.8244	0.9762	0.37164	29.7710
323	0.8193	0.9717	0.34325	25.9040
328	0.8142	0.9677	0.31711	22.7030
333	0.8089	0.9634	0.29671	19.9550
338	0.8037	0.959	0.27238	17.6190
343	0.7984	0.9545	0.22767	15.6140

a Standard uncertainties *u* are *u*(*ρ*) = 5 × 10^−4^ g cm^−3^, *u*(*η*) = 0.35%, *u*(*T*) = 0.02 K, and *u*(*p*) = 10 kPa.

**Fig. DB2:**
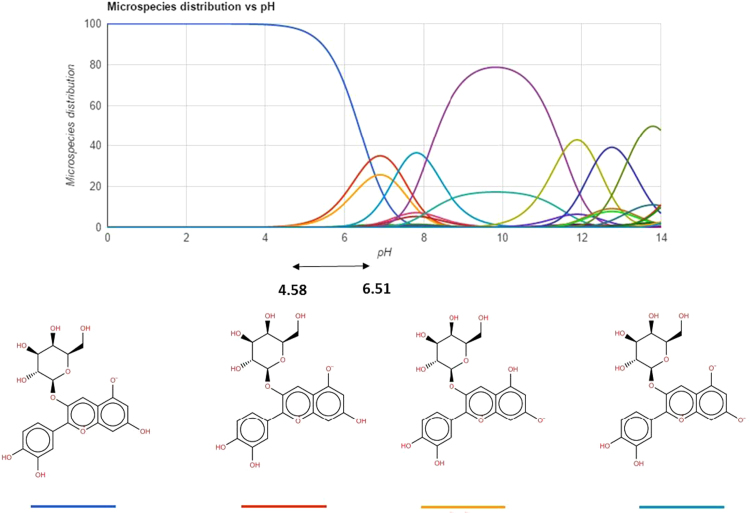
Chemical structure of cyanidin 3-O-glucoside at different pH values. This content was adapted from ChemSpider chemistry database [1].

**Fig. DB3:**
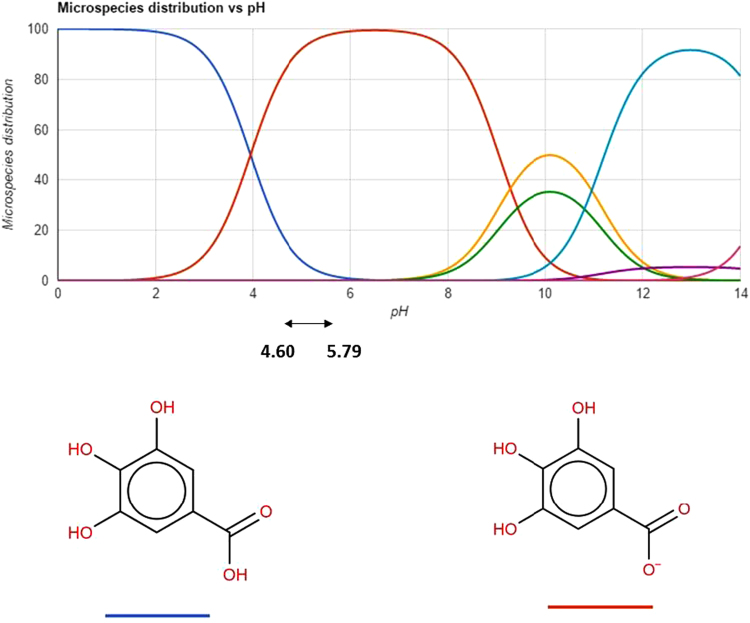
Chemical structure of gallic acid at different pH values. This content was adapted from ChemSpider chemistry database [1].

Table DB7 depicted the partition coefficients and extraction efficiency of phenol compounds, while the Fig. DB2, Fig. DB3DB4 present the speciation curve of cyanidin 3-O-glucoside, gallic acid and quercetin, respectively [1].

**Table DB7:** Partitioning coefficient and extraction efficiency of biocompounds at 298.15 K and 0.1 MPa.^a^

Biocompounds	PVP	K	EE
Cyanidin 3-O-glucoside chloride	10,000	11.12 ± 0.30	96.69 ± 0.07
	29,000	8.29 ± 0.18	95.61 ± 0.09
	40,000	1.32 ± 0.01	73.55 ± 1.44
Gallic acid	10,000	0.17 ± 0.01	32.00 ± 1.03
	29,000	0.22 ± 0.00	35.00 ± 0.00
	40,000	0.28 ± 0.01	41.00 ± 1.00
Quercetin	10,000	0.26 ± 0.01	41.48 ± 1.03
	29,000	0.26 ± 0.00	42.00 ± 0.09
	40,000	0.31 ± 0.01	42.97 ± 0.68

a Standard uncertainties *u* are *u*(*T*) = 1 K and *u*(*p*) = 10 kPa.

**Fig. DB4:**
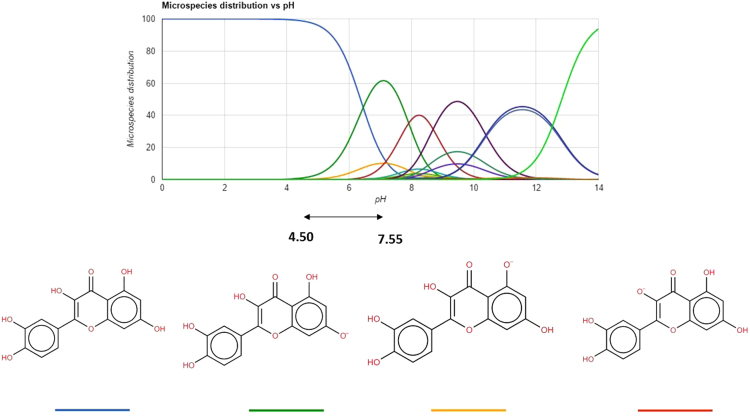
Chemical structure of quercetin at different pH values. This content was adapted from ChemSpider chemistry database [1].

## 2 Experimental design, materials, and methods

### 2.1 Phase diagrams

Aqueous solution of PVP (20–39 wt%) and ACN (80 wt%) were prepared and used for the binodal curve determination at 298.15 ± 1.00 K and 0.10 ± 0.01 MPa by cloud point method [2]. The experimental data were determined gravimetrically, within an uncertainty of ± 10^−5^ g (Shimadzu AUW220D, Philippines). PVP or ACN aqueous solutions were added drop-wise to each other until the visual detection of a cloudy solution (biphasic region). At this point water was drop-wised until the detection of a clear and limpid solution (monophasic region). This protocol was performed under constant magnetic stirring (Tecnal TE-085, Piracicaba-SP, Brazil) and was repeated several times in order to obtain enough points for the construction of a liquid–liquid equilibrium binodal curve. The experimental data of binodal curves were correlated using Merchuck et al. [2] (Eq. (1)).

(1) $$\left[ACN]=A\times \exp\{B\times {\left[PVP\right]}^{0.5}-C\times {\left[PVP\right]}^{3}\right\}$$

Where [ACN] and [PVP] are respectively the percentages by weight of acetonitrile and polyvinylpyrrolidone, and *A*, *B* and *C* are constants obtained by regression.

A mixture point at the biphasic region of each ternary system was prepared, vigorously stirred, and allowed to reach equilibrium and phase separation, for a minimum of 12 h at 298.15 ± 1.00 K and 0.10 ± 0.01 MPa and used to build the tie-lines (TLs). After the equilibration step, the top and bottom phases were carefully separated and weighted within ± 10^−5^ g. Each individual TL was determined by the application of the lever-arm rule, which describes the relationship between the weight of the top phase and the overall system weight and composition. The determination of the TLs was then accomplished by solving the following system of four equations (Eqs. (2), (3), (4), (5)) for the four unknown values of [ACN]_T_, [ACN]_B_, [PVP]_T_ and [PVP]_B_.

(2) $${\left[\mathrm{ACN}\right]}_{T}=A\;\exp\{B\times {\left[\mathrm{PVP}\right]}_{T}^{0.5}-C\times {\left[\mathrm{PVP}\right]}_{T}^{3})\}$$

(3) $${\left[\mathrm{ACN}\right]}_{B}=A\;\exp\left\{(B\times {\left[\mathrm{PVP}\right]}_{B}^{0.5}-(C\times {\left[\mathrm{PVP}\right]}_{B}^{3})\right\}$$

(4) $${\left[ACN\right]}_{T}=\left({\left[ACN\right]}_{M}/\alpha \right)-\left(\left(1-\alpha \right)/\alpha \right){\left[ACN\right]}_{B}$$

(5) $${\left[PVP\right]}_{T}=\left({\left[PVP\right]}_{M}/\alpha \right)-\left(\left(1-\alpha \right)/\alpha \right){\left[PVP\right]}_{B}$$

Where the subscripts M, T and B correspond to the initial mixture, top and bottom phases, respectively. The value of *α* is the ratio between the mass of the top phase and the total weight of the mixture. The system solution results in the ACN and PVP concentration in the top and bottom phases, and thus, TLs can be simply represented.

### 2.2 Phenolic compounds partitioning

The liquid–liquid systems for the partitioning of phenolic compounds were prepared in graduated centrifuge tubes (15 mL) by weighing the appropriate amounts of ACN and PVP (10,000 g mol^−1^, 29,000 g mol^−1^ and 40,000 g mol^−1^) until 10 g of system. Three mixing points in the two-phase region were chosen (System A: 12.05 wt% + 62.05 wt%; System B: 9.02 wt% + 59.02 wt%, and; System C: 8.89 wt% + 58.89 wt% for PVP + ACN, respectively). Each systems individually contained 600 mg L^−1^ of gallic acid, 100 mg L^−1^ of quercetin or 0.2 mg L^−1^ cyanidin 3-O-glucoside chloride.

After the complete mixing of all components for a given mixture composition, each system was centrifuged at 2000 × *g* for 10 min at 298.15 ± 1.00 K (Hettich Universal 320R, Germany) to favor the phase separation, and then each tube was placed in a thermostatic bath (Marconi MA-127) at 298.15 ± 1.00 K for at least 12 h, to reach the equilibrium. The tubes were sealed to avoid the ACN vaporisation. The volume and weight of each phase was measured and both phases were further separated for the quantification of each phenolic compound and for the determination of their pH values (phmeter DM-22 Digimed). At least three independent replicates were made and the average partition coefficients and associated standard deviations were therefore determined.

The concentrations of each phenolic compounds at top and bottom phase were quantified through UV-spectroscopy, using a Varian Cary 50 Bio UV–vis spectrophotometer, and at a wavelength of 435 nm (gallic acid), 690 nm (quercetin) and 525 nm (cyanidin 3-O-glucoside chloride) using a calibration curve previously established.

The partition coefficient (*K*) was defined as ratio between the phenolic compound concentration in the top (*C*_T_) and bottom phase (*C*_B_), as describe by Eq. (6).

(6) $$K=\frac{C_{T}}{C_{B}}$$

The extraction efficiency in the top (EE) was evaluated using Eq. (7):

(7) $$\mathrm{EE}=\frac{KR_{V}}{1+KR_{V}}$$

(8) $$R_{V}=\frac{V_{T}}{V_{B}}$$

where *R*_V_ is the ratio between the volumes of the top (*V*_T_) and bottom (*V*_B_) phase.
