# Supplementary material for: Liquid-Liquid extraction of phenolic compounds in systems based on acetonitrile + water + polyvinylpyrrolidone at 298.15 K
Source: Data Brief. 2018 Sep 27;20:2045–53. doi: 10.1016/j.dib.2018.09.067 (PMC6178207; doi:10.1016/j.dib.2018.09.067)
Supplement: Supplementary file 1 — Supplementary material [file mmc1.docx]

"Conflict of Interest

There is no conflict of interest in this paper. Declarations of interest: none"
